# Reduction of [^11^C](+)3-MPB Binding in Brain of Chronic Fatigue Syndrome with Serum Autoantibody against Muscarinic Cholinergic Receptor

**DOI:** 10.1371/journal.pone.0051515

**Published:** 2012-12-11

**Authors:** Shigeyuki Yamamoto, Yasuomi Ouchi, Daisaku Nakatsuka, Tsuyoshi Tahara, Kei Mizuno, Seiki Tajima, Hirotaka Onoe, Etsuji Yoshikawa, Hideo Tsukada, Masao Iwase, Kouzi Yamaguti, Hirohiko Kuratsune, Yasuyoshi Watanabe

**Affiliations:** 1 Department of Physiology, Osaka City University Graduate School of Medicine, Abeno-ku, Osaka, Japan; 2 Central Research Laboratory, Hamamatsu Photonics KK, Hamakita, Shizuoka, Japan; 3 Molecular Imaging Frontier Research Center, Hamamatsu University School of Medicine, Hamamatsu, Japan; 4 RIKEN Center for Molecular Imaging Science (CMIS), Kobe, Hyogo, Japan; 5 Psychiatry, Department of Clinical Neuroscience, Osaka University Graduate School of Medicine, Suita, Japan; 6 Department of Health Sciences, Faculty of Health Sciences for Welfare, Kansai University of Welfare Sciences, Kashiwara, Japan; University of Jaén, Spain

## Abstract

**Background:**

Numerous associations between brain-reactive antibodies and neurological or psychiatric symptoms have been proposed. Serum autoantibody against the muscarinic cholinergic receptor (mAChR) was increased in some patients with chronic fatigue syndrome (CFS) or psychiatric disease. We examined whether serum autoantibody against mAChR affected the central cholinergic system by measuring brain mAChR binding and acetylcholinesterase activity using positron emission tomography (PET) in CFS patients with positive [CFS(+)] and negative [CFS(−)] autoantibodies.

**Methodology:**

Five CFS(+) and six CFS(−) patients, as well as 11 normal control subjects underwent a series of PET measurements with *N*-[^11^C]methyl-3-piperidyl benzilate [^11^C](+)3-MPB for the mAChR binding and *N*-[^11^C]methyl-4-piperidyl acetate [^11^C]MP4A for acetylcholinesterase activity. Cognitive function of all subjects was assessed by neuropsychological tests. Although the brain [^11^C](+)3-MPB binding in CFS(−) patients did not differ from normal controls, CFS(+) patients showed significantly lower [^11^C](+)3-MPB binding than CFS(−) patients and normal controls. In contrast, the [^11^C]MP4A index showed no significant differences among these three groups. Neuropsychological measures were similar among groups.

**Conclusion:**

The present results demonstrate that serum autoantibody against the mAChR can affect the brain mAChR without altering acetylcholinesterase activity and cognitive functions in CFS patients.

## Introduction

Neurotransmission at the muscarinic cholinergic receptor (mAChR) in the central nervous system is involved in cognitive function [1]–[3], motor control [4], [5], and rapid eye movement sleep [6]. Abnormalities of the central mAChR system in Alzheimer’s disease correlate well with the degree of dementia [7]–[9]. Postmortem studies have shown that reductions in central mAChR systems were present not only in Alzheimer’s-type dementia [10], [11] but also in Huntington’s disease [12]–[14], Parkinson’s disease [15], and schizophrenia [16]–[18]. Five subtypes of mAChR, M_1–5_, have been identified by molecular cloning [19], and the M_1_ receptor has a significant role in cognitive function [3], [20]. These results suggest that the activity of the mAChR M_1_ plays a role in maintenance of cognitive function in neuropsychiatric diseases.

In recent years, numerous brain-reactive antibodies have been identified in human sera and have been proposed to relate to neurological or neuropsychiatric symptoms [21]–[23]. Even when antibodies are present in serum, the blood-brain barrier (BBB) prevents an influx of antibodies into the brain tissues in the healthy condition. In contrast, BBB compromise permits the influx of antibodies into the brain and induces neuropsychiatric symptoms in experimental animals [24].

Chronic fatigue syndrome (CFS) is a heterogeneous disorder characterized by persistent fatigue accompanied by rheumatologic, cognitive, and infectious-appearing symptoms [25], [26]. CFS research showed abnormal cytokine levels including tumour necrosis factor, interleukin-1, interleukin-6 [27], increased markers of inflammation [28] and stressful life events prior to CFS onset [29], [30]. It has been established through *in vitro* and *in vivo* studies that BBB function was disrupted by tumour necrosis factor, interleukin-1 and interleukin-6 [31]–[34]. The BBB is also impaired by local inflammation [35] and stress [36]. Therefore, CFS patients might have some BBB impairment. Increased levels of the serum autoantibody against the mAChR M_1_ have been reported in CFS patients [37].

These lines of evidence led us to investigate the effect of autoantibody against the mAChR on the muscarinic cholinergic system in the brain *in vivo*. The mAChR was evaluated using a positron emission tomography (PET) ligand *N*-[^11^C]methyl-3-piperidyl benzilate ([^11^C](+)3-MPB) [38], and acetylcholinesterase (AChE) activity was assessed with *N*-[^11^C]Methyl-4-piperidyl acetate ([^11^C]MP4A) [39]–[41].

## Materials and Methods

### Ethics

All subjects gave their written, informed consent to participate in the present study. The study was approved by the Ethics Committee of Osaka City University Graduate School of Medicine (Osaka, Japan) and Hamamatsu Medical Center (Hamamatsu, Japan).

**Table 1:** Demographic overview of control and CFS patients.

Variable	Control	CFS(−)	CFS(+)
N	11	6	5
Sex (female/male)	5/6	3/3	3/2
Age (years)	32.9±6.5	32.0±2.5	39.2±7.0
Extent of fatigue expressed by visual analogue scale, mean ± SD	1.1±0.7	6.7±1.4***	5.9±1.2***

Data are expressed as mean ± SD.

*** p<0.001, significantly different from the corresponding values for the control (one way ANOVA using a post hoc Student-Newman-Keuls test).

### Objective

The aim of the present study was to investigate the effect of serum autoantibody of mAChR on brain functions in CFS by comparing the central cholinergic system among CFS patients with positive (CFS(+)) and negative (CFS(−)) autoantibody against the mAChR.

### Participants

The serum samples from CFS patients were assayed for the autoantibody against the mAChR. All CFS patients included in this study were diagnosed according to the clinical diagnostic criteria [26] at Osaka City University Hospital (Osaka, Japan). Patients were divided into CFS(+) and CFS(−) groups according to the assay for the autoantibody against the mAChR (described below). Five CFS(+) patients (3 female and 2 male, 39.2±7.0 years old), 6 CFS(−) patients (3 female and 3 male, 32.0±2.5 years old), and 11 healthy controls (5 female and 6 male, 32.9±6.5 years old) took part in the PET study (Table 1). All study participants were Asian. All the patients were medicated with vitamin C and the Chinese herbal medicine hochuekkito. There is no evidence that vitamin C and hochuekkito affect mAChR in the brain. The exclusion criteria for study participation were smoking, drinking alcohol regularly and taking medications known to affect the central cholinergic system including AChE inhibitors. Control subjects were neurologically and psychiatrically normal and had no history of medication or drug or alcohol dependence.

### Description of Procedures

Serum samples were collected on the PET experimental day, and assayed for the autoantibody against the mAChR again to confirm the reliability of the immunoassay. After the acquisition of magnetic resonance images (MRI), PET experiments were performed. On the same day, patients filled out a questionnaire about the extent of fatigue [using a by the visual analogue scale] [42]. All participants underwent comprehensive neuropsychological tests. Predictive IQ was assessed by the Japanese version of the National Adult Reading Test [43]. Measurements of executive functions were obtained with the Wisconsin Card Sorting Test [44] and Advanced Trail-Making Tests, which was a touch panel version of the original trail-making test [45]. The Advanced Trail-Making Tests have been used as a task to measure mental fatigue [46], [47]. Non-verbal long term memory was assessed by the Rey Complex Figure test [48]. Finally, memory function was assessed comprehensively by the full version of the Japanese Wechsler Memory Scale-Revised [49].

### Detection of Autoantibody to mAChR by Radioligand Assay

The radioligand assay was conducted according to methods described in our previously published study [37]. The open reading frame of mAChR was obtained by reverse transcript polymerase chain reaction (PCR) amplification using poly-A RNA from the human hippocampus (CLONTECH Laboratories, Palo Alto, CA) as a template. The first strand cDNA was synthesized using ReverTraAce (TOYOBO, Tokyo, Japan) with random hexamers according to the manufacturer’s instructions. PCR using the following primer pairs containing either an EcoRI or an XhoI site. 5′-GGAATTCATGAACACTTCAGCCCCACCTGC-3′ and 5′-CCGCTCGAGTCAGCATTGGCGGGAGGGAGT-3′ (the EcoRI and XhoI sites have been underlined) was used. PCR was carried out using KOD-plus (TOYOBO) as a DNA polymerase. Each cDNA was digested with an EcoRI and an XhoI and ligated into the pET28a(+) expression vector (Novagen, Madison, WI). The [^35^S]-methionine-labeled protein was produced using cDNA, TNT Quick coupled Transcription/Translation System (Promega, Madison, WI), and [^35^S]-methionine (Amersham Biotech, Arlington Heights, IL) according to the manufacturer’s instructions. The [^35^S]-methionine-labeled protein was then applied to a Nick column (Amersham Biotech) to remove free [^35^S]-methionine, electrophoresed to SDS-PAGE (15% polyacrylamide gel), and autoradiography demonstrated the presence of a band component for the mAChR.

The [^35^S]-labeled human mAChR protein was diluted to 1000 counts per minute (cpm) per microliter by reaction buffer (50 mmol/l Tris-HCl, 150 mmol/l NaCl, 0.1% BSA, 0.1% Tween-20, and 0.1% NaN_3_, pH 7.4) and stored at −80 C until use. Ten microliters of a patient’s sera were diluted with 490 µl of reaction buffer. Thirty microliters of diluted patient sera and 20 µl of reaction buffer containing 20,000 cpm of [^35^S]-labeled human mAChR protein were incubated overnight at 4°C. The final dilution of each serum sample was 1∶50. The reaction mixtures were transferred to each well in a 96-well filtration plate (Millipore, Benford, MA), which had been pretreated with blocking buffer (50 mmol/l Tris-HCl, 150 mmol/l NaCl, 3% BSA, and 0.1% NaN_3_, ph 7.4) at 4°C overnight. Ten microliters of 50% protein G Sepharose 4FF (Amersham Bioscience) was added to each well to isolate the immune complex and then incubated for 45 min at room temperature. The plate was washed 10 times with 200 µl washing buffer (50 mmol/l Tris-HCl, 150 mmol/l NaCl, and 1% Tween-20, pH 7.4) using a vacuum manifold (Millipore). The filter was dried and OptiPhase SuperMix (Perkin-Elmer Life Science, Boston, MA) was added to each well before the quantity of precipitated labeled protein was counted in a 1450 MicroBeta TriLux apparatus (Perkin-Elmer Life Science). All samples were measured in duplicate. The inter-assay coefficient of variation varied from 6.3% to 9.6%.

The results were expressed as an antibody index and were calculated as follows:
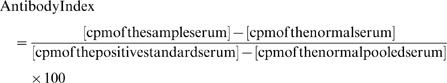



Commericial antibodies to human mAChR M_1_ (C-20) (Santa Cruz Biotechnology, Santa Cruz, CA) was used as the positive standard for anti-mAChR antibody. The cut-off value was calculated as the mean±2 S.D. in healthy controls.

### MRI and PET Experiments

MRI with 3D mode data acquisition was performed on a 3.0-T scanner (MRP7000AD, Hitachi, Tokyo, Japan) to determine the brain areas for setting the regions of interests (ROIs). MRIs from each subject revealed no apparent morphological abnormalities.

We used [^11^C](+)3-MPB to evaluate the activity of brain mAChR in the present PET study. In 1998, a human PET study with [^11^C](+)3-MPB had already been carried out under the approval of the local committee of the prefectural Research Institute for Brain and Blood Vessels in Akita [50]. In 2004, the Ethics Committee of Hamamatsu Medical Center approved our PET study with [^11^C](+)3-MPB, based on the approval of the human study performed by Takahashi and colleagues in a public facility. After the approval, we performed the current human PET study from 2004 to 2010, during which we tried hard to seek for patients with our criteria. In 2011, we planned another PET study with [^11^C](+)3-MPB in collaboration with other groups, and the collaborators requested us to re-examine the safety of (+)3-MPB because they wondered if the first precursor of [^11^C](+)3-MPB we had used in the human study was good enough to be used in their study. So, we asked Nard Institute Ltd to do the safety test (study number CG11117), and confirmed the safetiness.

PET was performed as described previously [42] on a brain SHR12000 tomograph (Hamamatsu Photonics KK, Hamamatsu, Japan) having an intrinsic resolution of 2.9×2.9×3.4 scanner in full width at half maximum, 47 slices, and a 163-mm axial field of view. Two PET measurements using [^11^C](+)3-MPB and [^11^C]MP4A were performed sequentially at 3-hour intervals on the same day. The order of [^11^C](+)3-MPB and [^11^C]MP4A PET measurements were counterbalanced across subjects. The specific radioactivities of these ligands were found to be more than 50 GBq/µmol after synthesis of [^11^C](+)3-MPB and [^11^C]MP4A, respectively.

After head fixation using a thermoplastic face mask, a 10-min transmission scan for attenuation correction was obtained. After a bolus injection of [^11^C](+)3-MPB (348.9±57.2 MBq), serial PET scans were performed with a total duration of 92 min (4×30 sec, 20×1 min, and 14×5 min). After a bolus injection of [^11^C]MP4A (297.6±53.8 MBq), serial PET scans were performed for a total of 62 min (4×30 sec, 20×1 min, and 8×5 min).

**Figure 1:**
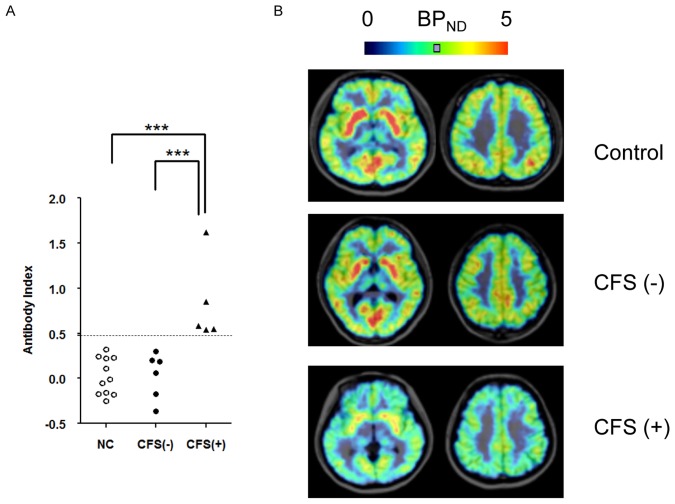
Serum autoantibody and PET images with [^11^C](+)3-MPB among normal control (NC) and CFS(−) and CFS(+) patients. (A) Antibody index against the muscarinic cholinergic receptor (mAChR) in serum from NC, CFS(−) and CFS(+) groups. ***p<0.001, significantly different from the corresponding value for the CFS(+) patients (one way ANOVA using a post hoc Student-Newman-Keuls test). (B) Representative parametric PET images of [^11^C](+)3-MPB binding in NC, CFS(−) and CFS(+) groups.

**Table 2:** Results of neuropsychological tests.

Test	Control	CFS(−)	CFS(+)
Japanese version of the National Adult Reading Test	72.0±11.1	79.3±13.7	75.4±9.3
Advanced Trail-Making Test A	124.3±28.7	125.1±19.2	125.5±30.7
Advanced Trail-Making Test B	163.2±34.6	161.5±31.2	187.4±70.1
Advanced Trail-Making Test C	263.9±45.0	260.8±45.8	275.7±27.0
Rey Complex Figure: immediate recall	28.7±2.5	29.8±2.7	28.7±3.3
Rey Complex Figure: delayed recall	28.0±3.3	28.7±3.1	27.8±2.7
Wisconsin Card Sorting Test	327.5±108.1	314.7±41.7	338.2±106.8
Wechsler Memory Scale-Revised			
General memory	112.1±8.0	114.5±9.4	109.2±5.3
Delayed memory	113.8±9.0	115.0±11.2	112.4±6.5
Verbal memory	109.7±9.1	112.0±10.6	107.0±7.0
Visual memory	114.0±5.5	114.7±5.2	113.2±6.3
Attention	102.0±14.6	100.5±16.6	103.8±13.5

Data are expressed as mean ± SD.

No significant differences were observed among control, CFS(−), and CFS(+) patients.

### PET Data Analysis

The brain MRI was first co-registered to the PET image by pixel-wise kinetic modeling software (Pixel-Wise Kinetic Modeling Group, Zurich, Switzerland). The following ROIs were drawn bilaterally on the registered MR images; dorsolateral prefrontal cortex, anterior cingulate cortex, amygdala, occipital cortex, parietal cortex, temporal cortex, orbitofrontal cortex, thalamus and cerebellum. These ROIs were then transferred onto the corresponding dynamic [^11^C](+)3-MPB images and static [^11^C]MP4A image.

For [^11^C](+)3-MPB analysis, the Logan reference tissue method was used in pixel-wise kinetic modeling software. In this study, the cerebellum was used as the reference region [51]. The Logan reference tissue method allows the estimation of the distribution volume ratio (DVR), which can be expressed as follows [52]:
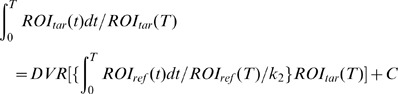
where ROI_tar_ and ROI_ref_ are the radioactivity concentrations of the target and reference region, respectively, at time-T. The DVR is the slope and k_2_ is the clearance rate from the reference region. A k_2_ value of 0.31 was used, according to a previous study [51]. C is the intercept of the Y-axis. The DVR is the ratio of the distribution volume in the target to the reference region. DVR minus one was calculated as BP_ND,_ which is the ratio at equilibrium of specifically bound radioligand to that of nondisplaceble radioligand (ND) in tissue [53]. Data recorded during the first 15 min were excluded based upon our previous PET study [38]. We also generated parametric images of the binding potential (BP_ND_) by the Logan reference tissue method based on pixel-wise kinetic modelling [54].

For [^11^C]MP4A analysis, the summation image from 32–62 min postinjection was obtained, and the uptake values in target ROIs divided by the uptake of the cerebellar hemisphere was used for the AChE activity ([^11^C]MP4A index) [55], [56].

### Statistics

The age, extent of fatigue, results of neuropsychological tests, and regional BP_ND_ values or uptake were compared among 3 groups with one way ANOVA using a post hoc Student-Newman-Keuls test. Statistical significance was set at P<0.05.

## Results

Figure 1A shows the radioligand assay in serum samples collected on the PET experiment day. There were 5 positive patients (CFS(+)) whose serum autoantibody was higher than the cut-off value shown as a dashed line. In normal controls, there were no subjects with positive autoantibody against the mAChR. As shown in Table 1, fatigue scores, expressed by visual analogue scale, were similar between CFS(+) and CFS(−) patients (5.9±1.2 vs. 6.7±1.4, respectively). In all the neuropsychological assessments, there were no significant differences among the 3 groups (Table 2).

Representative maps of the BP_ND_ of [^11^C](+)3-MPB using the Logan plot with reference regions are presented in Figure 1B. The BP_ND_ of [^11^C](+)3-MPB in each brain of CFS(+) patients were significantly lower than those in CFS(−) patients and control subjects (Fig. 1B, Table 3). Compared with controls, a 10–25% reduction of BP_ND_ was observed in CFS(+) patients (Table 3). AChE activity did not differ among the 3 groups (Table 3). There were no significant differences in BP_ND_ between CFS(−) patients and control subjects. There were no regions in which the BP_ND_ of [^11^C](+)3-MPB significantly correlated with any neuropsychological indices.

**Table 3:** Comparisons of [^11^C](+)3-MPB BP_ND_ and [^11^C]MP4A index among control, CFS(−) and CFS(+) groups.

	[^11^C](+)3-MPB BP_ND_			
ROI	Control	CFS(−)	CFS(+)	Reduction (%)
Dorsolateral Prefrontal Cortex	2.74±0.40	2.65±0.20	2.19±0.29* #	20
Anterior Cingulate Cortex	2.87±0.39	2.93±0.37	2.26±0.50* #	21
Orbitofrontal Cortex	2.71±0.30	2.79±0.21	2.14±0.35** ##	21
Temporal Cortex	2.57±0.24	2.61±0.22	2.24±0.28* #	13
Parietal Cortex	2.47±0.33	2.66±0.19	2.12±0.29* #	14
Occipital Cortex	2.60±0.28	2.69±0.27	2.25±0.24* #	14
Striatum	4.73±0.60	4.70±0.50	3.69±0.75* #	22
Thalamus	1.73±0.18	1.82±0.10	1.51±0.22* #	13
Amygdala	2.20±0.36	2.32±0.22	1.66±0.19** ##	25
Brainstem	0.95±0.15	0.92±0.16	0.86±0.15* #	10
	[^11^C]MP4A index			
Dorsolateral Prefrontal Cortex	0.43±0.04	0.43±0.03	0.44±0.02	−4
Anterior Cingulate Cortex	0.50±0.05	0.51±0.05	0.49±0.02	1
Orbitofrontal Cortex	0.49±0.05	0.47±0.04	0.47±0.03	3
Temporal Cortex	0.42±0.03	0.43±0.03	0.43±0.02	−2
Parietal Cortex	0.38±0.03	0.39±0.03	0.40±0.01	−6
Occipital Cortex	0.37±0.03	0.38±0.03	0.38±0.02	−4
Striatum	−	−	−	−
Thalamus	0.82±0.07	0.77±0.07	0.85±0.04	−4
Amygdala	0.64±0.05	0.61±0.07	0.64±0.04	1
Brainstem	0.84±0.06	0.87±0.07	0.82±0.05	2

Data are expressed as mean ± SD.

* p<0.05,

** p<0.01, significantly different from the corresponding values for the control.

# p<0.05,

## p<0.01, significantly different from the corresponding values for the CFS(−).

Reduction (%) reflects the extent of decreased in the rates of [^11^C](+)3-MPB BP_ND_ or [^11^C]MP4A index from control to CFS(+).

## Discussion

Reduction of [^11^C](+)3-MPB binding was observed in CFS(+) patients who showed a higher level of serum autoantibody against the mAChR, compared with CFS(−) patients and normal controls. In contrast, the AChE activity was similar in subjects from the 3 groups. The indices of intelligence and cognitive function did not differ among the 3 groups, and these indices did not relate to [^11^C](+)3-MPB binding in this study. To our knowledge, this is the first PET study to demonstrate a reduction of neurotransmitter receptor binding in brains of CFS patients with high levels of serum autoantibody. The present results suggest the possibility of the autoantibody interacting directly with the mAChR in the brain, although the autoantibody at this level did not affect cognitive function in CFS patients. The present finding supports the idea that penetration of the antibody into the brain resulted in impaired BBB function. This may be one possible mechanism by which the serum autoantibody could affect central mAChR function [57].

Although the precise mechanism of the production of the autoantibodies against the mAChR in the CFS brain is unclear, there are the following mechanisms based on an autoimmune reaction theory: 1) a viral infection of the brain tissue exposes the brain to self-antigen; and 2) an infection (not necessarily in the brain tissue) causes production of antibodies which, as a result of molecular mimicry, identify brain antigens as non-self and cause autoimmune reactions [58]. These mechanisms are plausible because a series of viruses such as the Epstein-Barr virus, human herpes virus 6, group B coxsackie virus, human T-cell lymphotrophic virus II, hepatitis C, enteroviruses and retroviruses were found to act as etiological agents for CFS [59]. Therefore, it is very likely that autoantibodies develop in some populations of CFS patients.

There are some possible reasons for the reduction of [^11^C](+)3-MPB binding in CFS(+) patients. First, the autoantibody may have penetrated through the impaired BBB directly destroying the mAChR in the brain. A second possibility is that increased endogenous acetylcholine (e.g. resulting from inhibition of AChE activity) competes with [^11^C](+)3-MPB at the mAChR. However, the latter seems unlikely. Our previous PET study showed that [^11^C](+)3-MPB did not compete with endogenous acetylcholine because of its high affinity for the receptors [60]. In addition, the present results indicate no significant changes in AChE activity assessed with [^11^C]MP4A, even in CFS(+) patients. A third possible mechanism underlying reduced [^11^C](+)3-MPB binding is that antibodies may act as receptor agonists or antagonists [21]. It was reported that serum autoantibodies against the mAChR displayed agonist-like activity, such as increased cGMP production, activated phosphoinositide turnover, and translocated protein kinase C [61]. All of these biological effects resemble the effects of the mAChR agonists like pilocarpine, and were minimized by the mAChR antagonist pirenzepine. In addition, the agonistic activity by these autoantibodies might induce desensitization, internalization and/or intracellular degradation of the mAChR, resulting in a progressive decrease of the mAChR expression in the brain [62]. Taken together, the present results suggest that autoantibodies penetrating the BBB from the serum to the brain may act on the mAChR directly and specifically in the CFS brain without altering AChE activity.

Five subtypes of mAChR, M_1–5_, have been identified by molecular cloning [19]. M_1_, M_2_ and M_4_ receptors are predominant subtypes expressed in different percentages among brain regions. Quantitative immunoprecipitation study indicates that the distribution percentages of M_1_, M_2_ and M_4_ receptors are 60%, 20% and 20% in the cortex, respectively. In the striatum, their distribution percentages are 30%, 20% and 50%, respectively [63]. We had expected a greater reduction of [^11^C](+)3-MPB BP_ND_ in the cortex than in the striatum because serum autoantibody detected in the present study was specific for the M_1_ receptor. However, similar reductions in the rate of [^11^C](+)3-MPB BP_ND_ were observed between the cortex and striatum (Table 3). One possible explanation for this is the low selectivity of [^11^C](+)3-MPB to the subtype of mAChR. The *Ki* values of (+)3-MPB for the human receptors from M_1_ to M_5_ were 1.34, 1.17, 2.82, 1.76, and 5.91 nM, respectively, as assessed with five cloned human mAChR subtypes expressed in CHO-K1 cells (unpublished data). These data indicate that the M_1_ receptor and the other subtype of mAChR contribute to the reduction in the rate of [^11^C](+)3-MPB BP_ND_ in CFS(+) patients.

Because the M_1_ receptor has a significant role in cognitive function [3], [20], we predicted cognitive impairment in CFS(+) patients. However, cognitive function in CFS patients was not associated with changes in [^11^C](+)3-MPB BP_ND_. One plausible explanation is that reduction in the level of [^11^C](+)3-MPB BP_ND_ occurs within a range of preserved cognitive function. Indeed, we recently reported the relationship between [^11^C](+)3-MPB BP_ND_ and cognitive function in conscious monkeys, showing that there were thresholds (ca. 30–40% in cortex and ca. 20–30% in brainstem) of activity of the brain mAChR to induce cognitive impairment [64], [65].

### Limitations

We cannot exclude the possibility that the autoimmune reaction occurred as a secondary process to the reduction of the mAChR. In addition, our findings relate to a small subset of CFS patients. This was chiefly due to the difficulty in obtaining CFS patients’ consent to participate in the present study because it entailed a series of PET and MRI measurements, requiring a significant commitment of time from each subject. Additional experiments will be necessary to fully validate the present findings. Increases in the serum autoantibody against the mAChR have also been reported in Sjögren syndrome [66] and other psychiatric disorders including schizophrenia [61], [62], [67]. Therefore, our results cannot be generalized to the entire CFS population.

### Summary

Our results demonstrate the usefulness of PET as a tool for detecting a reduction of neurotransmitter receptor binding in the brains of patients with high levels of serum autoantibody. Further follow up studies on a number of CFS patients are required in order to more thoroughly investigate alterations in cholinergic and neuronal functions with regard to levels of mAChR autoantibody and clinical symptoms.
